# A Study of Proline Metabolism in Canola (*Brassica napus* L.) Seedlings under Salt Stress

**DOI:** 10.3390/molecules17055803

**Published:** 2012-05-16

**Authors:** Mubshara Saadia, Amer Jamil, Nudrat Aisha Akram, Muhammad Ashraf

**Affiliations:** 1 Department of Chemistry, University of Sargodha, Sargodha 4100, Pakistan; 2 Molecular Biochemistry Lab, Department of Chemistry and Biochemistry, University of Agriculture, Faisalabad 38040, Pakistan; 3 Department of Botany, Government College University, Faisalabad 38040, Pakistan; 4 Department of Botany, University of Agriculture, Faisalabad 38040, Pakistan

**Keywords:** canola, Δ^1^-pyrroline-5-carboxylate synthase1 (P5CS1), proline dehydrogenase (PDH), proline, salt tolerance

## Abstract

Expression analysis of crop plants has improved our knowledge about the veiled underlying mechanisms for salt tolerance. In order to observe the time course effects of salinity stress on gene expression for enzymes regulating proline metabolism, we comparatively analyzed the expression of specific genes for proline metabolism in root and shoot tissues of salt-tolerant (cv. Dunkled) and salt-sensitive (cv. Cyclone) canola (*Brassica napus* L.) cultivars through reverse-transcriptase polymerase chain reaction (RT-PCR); following the NaCl treatment for various durations. Both lines showed an increase in Δ^1^-pyrroline-5-carboxylate synthase1 (*P5CS1*) gene expression after induction of salt stress with enhanced expression in the root tissue of the tolerant line, while maximum expression was noted in the shoot tissues of the sensitive line. We observed a much reduced proline dehydrogenase (*PDH*) expression in both the root and shoot tissues of both canola lines, with more marked reduction of *PDH* expression in the shoot tissues than that in the root ones. To confirm the increase in *P5CS1* gene expression, total proline content was also measured in the root and shoot tissues of both the canola lines. The root tissues of canola sensitive line showed a gradually increasing proline concentration pattern with regular increase in salinity treatment, while an increase in proline concentration in the tolerant line was noted at 24 h post salinity treatment after a sudden decrease at 6 h and 12 h of salt treatment. A gradually increasing concentration of free proline content was found in shoot tissues of the tolerant canola line though a remarkable increase in proline concentration was noted in the sensitive canola line at 24 h post salinity treatment, indicating the initiation of proline biosynthesis process in that tissue of sensitive canola.

## 1. Introduction

Soil salinity is a prevalent abiotic stress that adversely affects crop productivity worldwide. Since the increased levels of sodium salts in soil has limited the agricultural productivity, it has been considered that the deficit of cultivable land due to salinity was likely to increase over the next 20 years, impinging on world food supplies [1]. The situation has been intensified more by global climate change adding more to the desertification and salinization. Therefore, there is definitely the need of upgrading drought and salinity tolerant crops [2]. 

Canola, after soybean and palm oil, is ranked as the third major source of edible oil [3]. Thus the higher demand has certainly led to increased canola acreage where some land was likely to suffer from salinity [4]. Farmers and consumers are affected economically by a reduction in yield and/or oil quality of important oilseed crops [5]. Though saline soils or soils irrigated with saline water present potential hazards to canola production and expansion [6], studies by Francois [4] described no effect of salinity on seed oil composition, although the seed yield was reduced significantly. Similarly, Qasim *et al.* [7] also reported the non-imperative effect of salt stress on canola seed oil and erusic acid contents.

In plant species, where there is a high rate of salt uptake, compartmentation through vacuoles exceeds the limits and the received salt mainly imposes additional stress on plants which ultimately affects their salt tolerance potential [8]. Compartmentation of Na^+^ by vacuoles was also responsible for lowered cell water potential and the sustained water absorption from the soil [9]. However, this lowered osmotic potential in the vacuole was balanced with that of the cytoplasm by accumulation of non-toxic (compatible) osmolytes in the cytosol [10]. In higher plants, the generally found compatible osmolytes are sugars (low in molecular weight), organic acids and polyols.The mainly distributedosmolyte considered in water-and salt-stressed plants was proline (Pro) [11,12]. In addition to reducing cytosolic osmotic potential, it plays a vital role in protein protection against denaturation [13] as well as in scavenging reactive oxygen species, ROS [14]. Pro biosynthesis adopts two routes: from the Glu (Glutamate) and/or Orn (Ornithine) pathways. However for Pro biosynthesis in osmotically stressed plants, Glu is the primary precursor rather than Orn [15]. In higher plants, the osmotic stress-induced accumulation of proline is dependent on the expression of the enzymes *Δ*^1^-pyrroline-5-carboxylate synthase (*P5CS*) and proline dehydrogenase (*PDH*) that catalyze the rate-limiting steps of proline biosynthesis and degradation, respectively. To understand the molecular mechanism of proline accumulation in *Brassica napus* (canola), Xue *et al*. [16] isolated and characterized the cDNAs encoding *Δ*^1^-pyrroline-5-carboxylate synthetase (*BnP5CS*), ornithine *Δ*-aminotransferase (BnOAT) and proline dehydrogenase (*BnPDH*). The authors have reported stress-induced proline accumulation in *B. napus* due to the reciprocal action of activated biosynthesis and inhibited proline degradation. Furthermore, the response of sodium chloride stress in different spring canola cultivars has been recently studied by Toorchi *et al.* [17] who suggested an ample genetic variability among rapeseed genotypes which could be used in breeding programs. They found a significant increase in free proline contents in canola leaves with increase in external NaCl concentration. Similarly, Nazarbeygi *et al.* [18] also studied the response of canola to different levels of salinity and found a significant increase in proline content in leaf and root tissues. Abscisic acid (ABA) and salt stress induced stimulation of proline synthesis was observed through a potent activation of *P5CS1* expression and *PDH* inhibition to different amounts in shoots and roots of light-grown *Arabidopsis* plants [19]. However, the possible role(s) of proline accumulation under stressed conditions has been controversial. Proline and its related metabolites were presumed to serve as the signaling factors in stress-induced cell destruction [20,21,22]. Verbruggen and Hermans [23] have presented a debate on proline toxicity in plants in their review on proline accumulation in plants. Recently, Lv *et al*. [24] have noted an inverse relation of Pro accumulation and thermotolerance of *Arabidopsis* seedlings during heat stress, which was ascribed to increased ROS production via the Pro/P5C cycle and inhibition of ABA and ethylene biosynthesis.

Here, we assume that the differential expression of genes controlling proline metabolism in canola varies with the time of exposure to salt stress. The objective of the present study was to assess the differential expression of genes regulating the proline metabolic processes using the Reverse Transcriptase reaction (RT-PCR) for salt tolerance in two canola lines differing in salt tolerance. The knowledge about proline expression profile, would allow the establishment of protective or toxicity indicator role of this key metabolite in canola that may help to produce a highly salt tolerant canola cultivar. 

## 2. Results

### 2.1. RT-PCR Analysis of Canola Gene Expression

**Figure 1:**
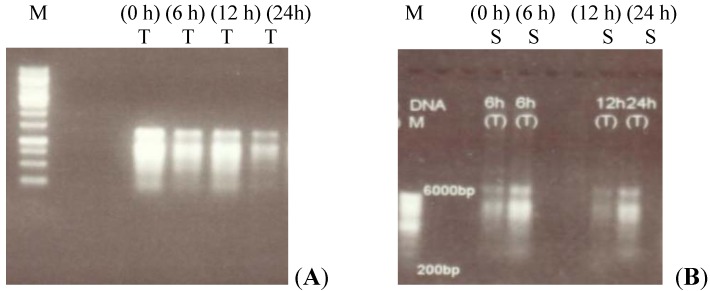
Representative total RNA extracted from root tissues of two canola lines differing in salt tolerance. (**A**) Total RNA isolated from root tissues of canola tolerant (T) line, harvested at 0, 6, 12 and 24 h after the induction of salt stress (control to salt treated plants). (**B**) Total RNA isolated from sensitive (S) canola line root tissues harvested at different time intervals. M is the 1 kb DNA ladder.

Total RNA was extracted from the control and NaCl-treated root and shoot tissues of the two canola lines, following NaCl treatment for various durations. The total RNA concentration was measured spectrophotometrically and then fractionated on a 1% agarose gel (Figure 1A,B).

To observe the expression of genes regulating proline metabolism, specific primers were used as described by Kant *et al*. [25] in their study on differential gene expression between *Thellungiella halophila* (halophyte) and *Arabidopsis thaliana* for higher levels of the compatible osmolyte, proline, and tight control of Na^+^ uptake in *T. halophila*. Primers designed from the various *A. thaliana* gene sequences were able to amplify similar-sized PCR products (~100 bp). Salt responsive cDNAs from canola young tissues treated with 150 mM NaCl treatment for 24 h were amplified via reverse transcription PCR (RT-PCR).

### 2.2. Pyrroline-5-Carboxylate Synthetase1 (P5CS1) Up-Regulation in Canola Shoots

**Figure 2:**
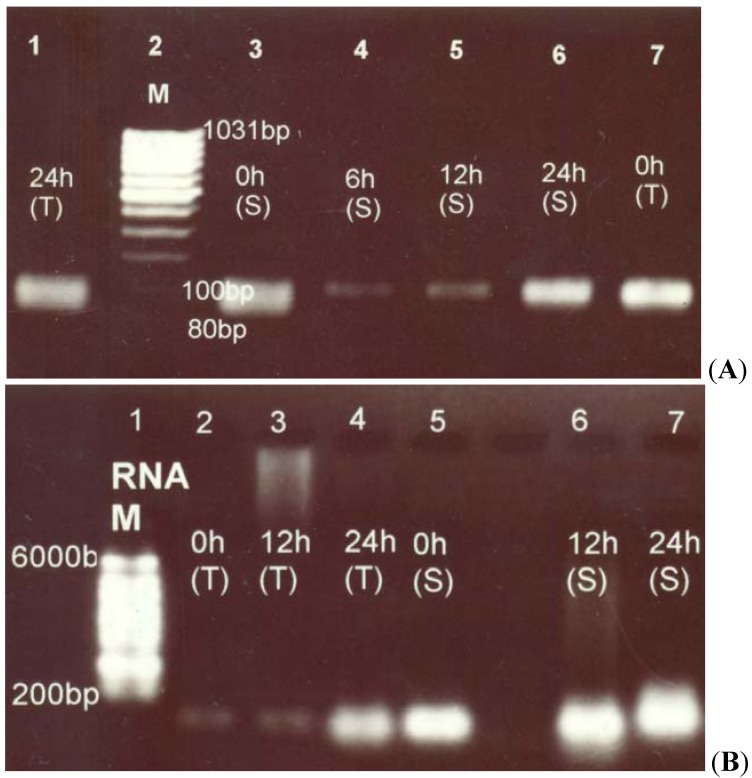
*Pyrroline-5-carboxylate synthetase 1 (P5CS1)* gene expression in root and shoot tissues of salt-tolerant and salt-sensitive canola lines. (**A**) *P5CS1* gene expression in root tissues of two contrasting canola lines at different time intervals after salt-treatment. (**B**) *P5CS1* gene expression at different time intervals in shoot tissues of two contrasting canola lines. M is the short range DNA marker with last band of 200 bp.

To test the increase in proline accumulation in plants under salt stress, we observed the expression of genes encoding proline biosynthesis and degradation enzymes. In our findings Pyrroline-5-carboxylate synthetase1 (*P5CS1*), catalyzing the 1st step in proline (Pro) biosynthesis [26], was induced by salt stress to a higher level in both canola lines with increased expression in the shoot tissues than in the root ones. Figure 2A,B show the *P5CS1* expression in root and shoot tissues of canola under salt stress at different time intervals respectively. 

The root tissue of the sensitive line showed the increased *P5CS1* expression after a sharp decrease at 6 h and 12 h of post-salinity treatment and reached maximum at 24 h of salinity induction, while equally enhanced *P5CS1* expression was noted in tolerant line. Whereas, in shoot tissue of sensitive canola line, a remarkable increase in *P5CS1* expression was noted, while a gradual increase in expression from 0 h to 24 h post-treatment was observed in the tolerant line.

### 2.3. Proline Dehydrogenase (PDH) Down-Regulation under Salt Stress

**Figure 3:**
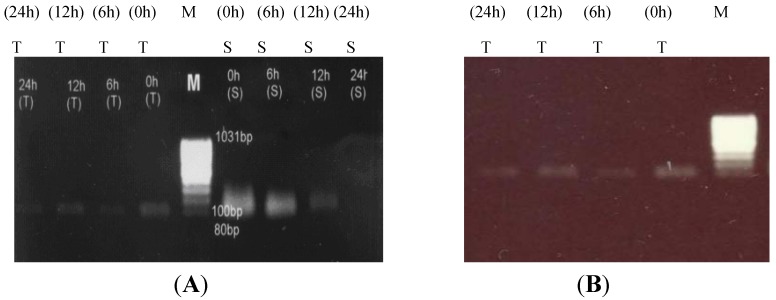
Proline dehydrogenase (*PDH*) gene expression in root and shoot tissues of salt-tolerant and salt-sensitive canola lines. (**A**) Proline dehydrogenase (*PDH*) gene expression in root tissues of two contrasting canola lines at different time intervals after the salinity treatment. (**B**) Proline dehydrogenase (*PDH*) gene expression in shoot tissue of two contrasting canola lines at different time intervals after salt-stress.

Under salt stress, proline accumulates in plant tissues as the major osmoprotectant. Proline dehydrogenase (PDH) catalyzes the rate limiting step in proline catabolism [27]. We observed that *PDH* expression was greatly reduced in canola shoot tissues as compared to that in root ones at different time intervals after the salinity shock imposed to the seedlings (Figure 3A). In roots of the sensitive canola line, *PDH* expression was observed in control (0 h) plants, but it decreased gradually after 6 h of salinity treatment. The tolerant plants showed a lower level of expression with gradually decreasing level of expression from 0 h to 24 h after imposing the salt treatment (Figure 3B).

### 2.4. Effect of Salt Stress on Proline Accumulation

**Figure 4:**
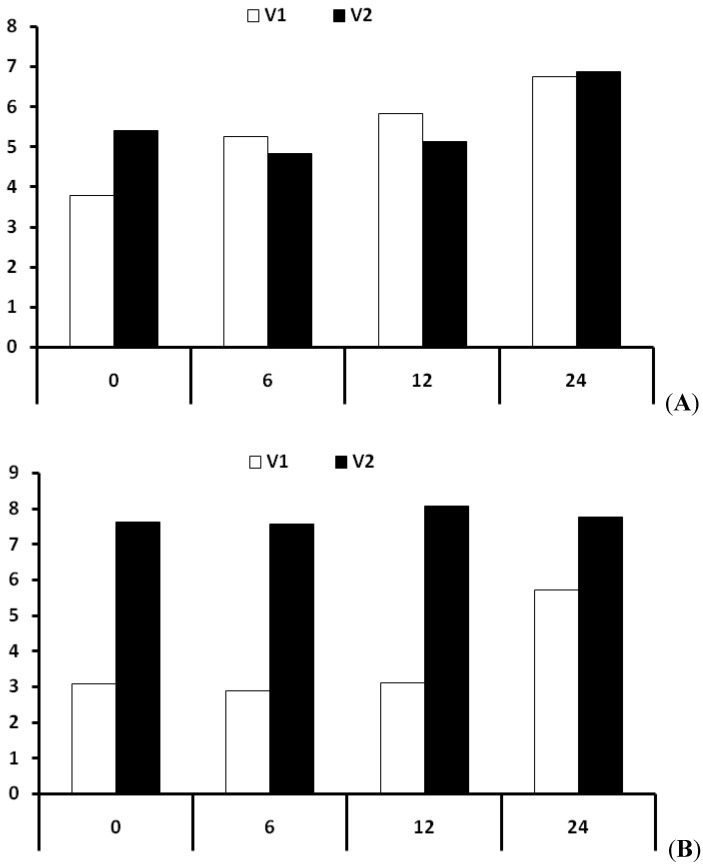
(**A**) Time-course studies of proline accumulation in root tissues of two contrasting canola cultivars after salt stress. The sensitive canola line (Cyclone) is represented by V1, whereas, the tolerant one is represented by V2 (Dunkled). Average of three determinations is presented with bars. (**B**) Time-course studies of proline accumulation in shoot tissues of two contrasting canola cultivars after salinity stress. The sensitive canola line (Cyclone) is represented by V1, whereas, the tolerant one is represented by V2 (Dunkled). Average of three determinations is presented with bars.

To confirm the increased *P5CS1* gene expression in canola seedlings, we also monitored the accumulation of proline, a reported stress-inducible metabolite. A remarkable time-dependent increase in free proline accumulation (up to 24 h of salinity treatment) was noticed in salinity-stressed seedlings of both canola cultivars. The root tissues of the canola sensitive line showed a gradually increasing proline concentration pattern with regularly increasing time intervals after the salinity treatment. However, an abrupt decrease in proline concentration was found in the tolerant canola line at 6 h and 12 h post salt treatment, however this concentration increased after 12 h of salinity treatment; 24 h post salinity treatment (Figure 4A). The shoot tissues showed progressively increasing free proline concentration in shoot tissues of the tolerant canola line. A remarkable increase in proline concentration was noted in the sensitive canola line at 24 h post salinity treatment (Figure 4B).

## 3. Discussion

### 3.1. Growth of Canola Cultivars under Saline Conditions

The salt-tolerant (Dunkled) and the salt-sensitive (Cyclone) canola cultivars were grown in a growth room under controlled conditions, irrigated with 150 mM NaCl in Hoagland’s solution at different time intervals (3 h, 6 h, 12 h and 24 h post salinity treatment), as mentioned earlier. We chose these time periods because in previous study, Taji *et al.* [28] had shown that *Thellungiella halophila* and *Arabidopsis thaliana* displayed increased NaCl uptake within the initial 12 h of salt stress and after 24 h *Arabidopsis* showed a marginally higher concentration of sodium. Similarly, *Arabidopsis thaliana* and *Thellungiella halophila* plants were exposed to NaCl stress for 3 h and 24 h; two time points [29]. They observed that in contrast to *Arabidopsis*, *Thellungiella halophila* (*Thellungiella salsuginea*; salt cress), displayed extreme tolerance to high salinity, low humidity and freezing, and at 150 mM NaCl, it maintained unimpeded growth. 

### 3.2. Comparative P5CS1 Expression

The comparative study of proline accumulation in the tolerant and the sensitive lines of canola plants was also necessary to correlate its accumulation with the gene expression studies of the enzymes involved in proline biosynthesis and degradation. Stress sensation and signal transmission results in several physiological and biochemical changes at the cellular level including production of several metabolites by triggering the induction of genes involved in their synthesis [5]. The rapidly increasing accumulation of this important metabolite in response to salt-stress was an indication that the plants were actively expressing the stress responses at the time when subjected to transcriptional profiling. The RT-PCR analysis showed in root tissue of tolerant line the higher level of *P5CS1* expression. The shoot tissue of salt-sensitive line showed a relatively higher, gradually increasing level of *P5CS1* expression (Figure 2A,B). The *P5CS1* expression induced by salt stress in shoot tissues relative to that in the root tissues has directed the consideration of initiation of proline biosynthesis in that tissue. Earlier it was demonstrated that roots were the important sites of proline synthesis, but export most of the product to shoot tissues [30]. In the present study, a sudden increase in *P5CS1* expression in root tissue of the sensitive line 12 h post the salinity treatment indicates a process of proline accumulation at this stage. In the shoot tissue of the salt-sensitive canola line, this increase was more profound. This may be contradictory to the hypothesis that more proline accumulates in tolerant plants. Therefore, the RT-PCR results have suggested a higher proline biosynthesis in the roots of the tolerant canola line while this occurs in the shoots of the sensitive canola line. 

### 3.3. Comparative PDH Expression

The very low level of expression of the gene encoding proline degradation enzyme, *PDH*, was found in the root and shoot tissues of both canola lines (Figure 3A,B). These findings showed the increased gene expression of enzyme involved in proline biosynthesis and consequently the decreased gene expression of enzyme that regulates proline degradation which ultimately leads to the increased accumulation of total proline content in canola plants. 

Proline metabolism was found to be modulated by differential regulation of organ specific expression of *PDH* and duplicated *P5CS* genes in *Arabidopsis* [19]. In *Arabidopsis,* proline was found to accumulate after salt stress (NaCl) attaining 20% of the total free amino acid pool in the presence of 0.75% NaCl [31]. We observed, as the expression level of the gene encoding the proline biosynthetic enzyme, *P5CS1*, has increased in the shoot tissues, there was an opposing decrease in the gene encoding the proline catabolic enzyme *PDH*, indicating the accumulation of proline in that tissue. The tolerant plants showed the relatively lower level of *PDH* expression in comparison to the sensitive canola plants. Here, the proline metabolite did not act as a toxicity indicator yet assumed a protective role. A correlation between proline synthesis stimulation by abscisic acid (ABA) and salt stress, and activation of *P5CS1* expression and *PDH* inhibition to different extent in shoots and roots of light-grown *Arabidopsis* plants has been observed [19]. Previously, it was found that the expression of *ProDH* was induced by rehydration after dehydration in *Arabidopsis* plants [32].

### 3.4. Comparative Total Proline Expression

Under stress conditions, many physiological processes of plant tissues (e.g., photosynthesis, stomatal conductance, and leaf expansion), are severely affected. The osmolyte accumulation in plant cells contributes via lowering the cell osmotic potential so that it can maintain the normal continuity of these processes [33]. Proline is considered to act as a regulatory or signaling molecule [34] in addition to being a reliable indicator of the environmental stress imposed on plants [35], and as an osmoregulator [12,13]. The evaluation of changes in proline content in different plant tissues was studied by different researchers. An increase in proline content was observed in both mature and young stressed leaves of sunflower (*Helianthus annuus* L. cv. Catissol 01) plants by Cechin *et al.* [33]. The young stressed leaves synthesized nearly seven times more proline than non-stressed leaves while the mature stressed leaves synthesized only four times more. The findings supported the positive role of proline as an osmoregulator, particularly in young leaves, which seems to play role in the survival mechanism for the plants under water stress [33]. Therefore different studies described the increase in proline content in root and shoot tissues with enhancement of salt [36,37] and drought stress [38].

We used the 14 day-old canola seedlings (Dunkeld: tolerant and Cyclone: sensitive) subjected to salt-stress treatment by supplementing their growth medium with 150 mM NaCl. This concentration (150 mM NaCl) has been used in several previous gene expression studies, as it was stated that this level of NaCl induces a moderate stress response rather than having a lethal effect on plant growth [39,40]. Total proline was found to increase in response to salt stress in both the cultivars; with a significant increase in the tolerant line. However, the gradual increase in proline expression in root tissues of the sensitive line definitely supports its role as an environmental toxicity indicator (Figure 4A). In shoot tissues of both the lines, it was observed that free proline accumulated in a slow growing manner reaching a maximum up to 24 h post salinity treatment (Figure 4B). This increase in expression was the indication of proline biosynthesis in shoot tissues. This is contrary to previous reports showing its synthesis only in roots and consideration that the higher proline concentration in shoot tissues was due to its rapid transportation to that tissue [30,41].

## 4. Experimental

### 4.1. Plant Material and Salt Treatments

Healthy seeds from two canola cultivars, Dunkled (salt-tolerant) and Cyclone (salt-sensitive), obtained from Ayub Agricultural Research Institute, Faisalabad, were geminated in washed moist sand in plastic pots in a controlled growth room at 22 °C with a 16/8 h light/dark photoperiod and light intensity of 150 μmol m^−2^s^−1^. The selected crop lines have already been reported for their differential salt tolerance, *i.e.*, cv. Dunkled is salt-tolerant, while cv. Cyclone is salt-sensitive [42]. Five-day-old seedlings were fertilized with full strength Hoagland’s nutrient solution [43]. Two-week-old seedlings were irrigated with 150 mM NaCl in Hoagland’s nutrient solution. Control plants remained in the nutrient solution. The seedlings were harvested after 3, 6, 12 and 24 h from the start of salt treatment to observe the effect of the salinity shock on proline gene expression. Plant samples (10 plants per sample) collected were frozen in liquid N_2_. 

### 4.2. Total RNA Isolation and Reverse Transcriptase-PCR

Total RNA was isolated from the shoot and root tissues using the plant RNeasy system (Qiagen, Missisauga, ON, Canada), following the manufacturer’s instructions. The total RNA was quantified with a Gene Quant Pro (Amersham Biosciences, Pittsburg, PA, USA) spectrophotometer and the quality was analysed by fractionating it on a 1% agarose gel [44]. The one step RT-PCR kit was used for cDNA synthesis as well as the PCR amplification of the isolated fragment according to the supplier’s (Novagen, Houston, TX, USA) instructions. This kit was preferred for gene expression analysis, as one step RT-PCR can replace methods for detecting and quantifying gene expression such as Northern blots, *in situ* hybridization, dot blots, S1 nuclease assays and conventional two steps RT-PCR (the two enzyme/two buffer system). Primers for amplification of PCR products between 50 to 120 bp were obtained from Genelink (Margate, NJ, USA), designed using *A. thaliana* sequences as listed by Kant *et al.* [25]. The sequences of each primer pair are as follows:

Δ*^1^***-*Pyrroline-5-carboxylate synthetase1 (P5CS1)*:**
  F 5^/^-GAGCTAGATCGTTCACGTGCTTT-3^/^
  R 5^/^-ACAACTGCTGTCCCAACCTTAAC-3^/^
  ***Proline dehydrogenase (PDH)*:**
  F 5^/^-TCACAACCACTGAGCTAAAGTGAGA-3^/^
  R 5^/^-CGATGACGCTGTATCTTGTGATG-3^/^

### 4.3. PCR Conditions and Analysis

RT-PCR was performed on a Perkin Elmer GeneAmp PCR system 2400 (Bloomfield Hills, MI, USA). The following program for the reaction was used: Reverse Transcription: 30 min at 60 °C; Initial PCR Activation: 2 min at 94 °C; Denaturation: 1 min at 94 °C; Annealing/ Extension: 90 sec at 60 °C repeat for 40 cycles and Final Extension: 7 min for 60 °C. For different primer pairs, an annealing temperature about 5 °C below the melting temperature (Tm) was chosen. For each PCR reaction, 1 μg of the total RNA was added to a mixture containing 5× reaction buffer, 2.5 mM dNTPs, 25 mM Mn(OAc)_2_, 10 pmol/μL of each gene-specific primer pair, 10 units/μL RNase inhibitor. To each tube, 5U *rTth* polymerase was added to a total volume of 50 μL reaction mixture. The results were analyzed on a 1% agarose gel after staining with ethidium bromide on gel documentation system (Cambridge, UK).

### 4.4. Total Proline Determination

Total proline was quantified using the method described by Bates *et al.* [45]. Fresh samples of whole seedlings were triturated in 10 mL of 3% sulfosalicylic acid. After extensive grinding, the homogenate was filtered using Whatman filter paper No.2. The filtrate (2.0 mL) was mixed with acid ninhydrin (2.0 mL, 1.25 g ninhydrin in 30 mL glacial acetic acid and 20 ml of 6 M orthophosphoric acid), and glacial acetic acid (2.0 mL) in a test tube. The mixture was incubated in a water bath at 100 °C for 60 min and then cooled in an ice bath. Toluene (4.0 mL) was added to the solution and mixed vigorously by passing a continuous stream of air for 1–2 min. The toluene containing the chromophore was aspirated from the aqueous phase, warmed at room temperature and the absorbance was noted at 520 nm on a UV-Visible spectrophotometer (IRMECO U2020, Geesthact, Germany) using toluene as a blank. The proline concentration was worked out from a standard curve as follows:



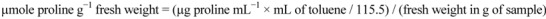



## 5. Conclusions

From the present study it has been concluded that *P5CS1* expression was induced by salt stress to a higher level in canola shoot tissues than in the root tissues. A process of proline accumulation was noticed in a sensitive canola line 24 h post salinity treatment which may be the osmotoxic indication represented by its accumulation in the salt-sensitive canola line. A relatively lower *PDH* expression was found in tolerant plants than in sensitive ones, which also indicated the osmoprotectant role of proline in those plants. Therefore, the exact role of this metabolite in canola plants is yet to be determined. Further investigations would be helpful in elucidating the role of proline in this regard as many salt-responsive genes do not contribute to tolerance; rather, their induction reflects salt stress damage. 
